# The Influence of HIV and Schistosomiasis on Renal Function: A Cross-sectional Study among Children at a Hospital in Tanzania

**DOI:** 10.1371/journal.pntd.0003472

**Published:** 2015-01-22

**Authors:** Neema M. Kayange, Luke R. Smart, Jennifer A. Downs, Mwanaisha Maskini, Daniel W. Fitzgerald, Robert N. Peck

**Affiliations:** 1 Department of Pediatrics, Weill Bugando School of Medicine, Catholic University of Health and Allied Sciences, Mwanza, United Republic of Tanzania; 2 Center for Global Health, Weill Cornell Medical College, New York, New York, United States of America; 3 Division of Infectious Diseases, Department of Medicine, Weill Cornell Medical College, New York, New York, United States of America; Rosetta Genomics, ISRAEL

## Abstract

**Background:**

Schistosomiasis and HIV are both associated with kidney disease. Prevalence and factors associated with abnormal renal function among HIV-infected children in Africa compared to uninfected controls have not been well described in a schistosomiasis endemic area.

**Methodology/Principal Findings:**

This cross-sectional study was conducted at the Sekou Toure Regional Hospital HIV clinic in Mwanza, Tanzania. A total of 122 HIV-infected children and 122 HIV-uninfected siblings were consecutively enrolled. Fresh urine was obtained for measurement of albuminuria and *Schistosoma* circulating cathodic antigen. Blood was collected for measurement of serum creatinine. Estimated glomerular filtration rate (eGFR) was calculated using the modified Schwartz equation. Renal dysfunction was defined operationally as eGFR<60mL/min/1.73m2 and/or albuminuria>20mg/L in a single sample. Among 122 HIV-infected children, 61/122 (50.0%) met our criteria for renal dysfunction: 54/122 (44.3%) had albuminuria>20mg/L and 9/122 (7.4%) had eGFR<60. Among 122 HIV-uninfected children, 51/122 (41.8%) met our criteria for renal dysfunction: 48/122 (39.3%) had albuminuria>20mg/L and 6/122 (4.9%) had eGFR<60. Schistosomiasis was the only factor significantly associated with renal dysfunction by multivariable logistic regression (OR = 2.51, 95% CI 1.46–4.31, p = 0.001).

**Conclusions/Significance:**

A high prevalence of renal dysfunction exists among both HIV-infected Tanzanian children and their HIV-uninfected siblings. Schistosomiasis was strongly associated with renal dysfunction.

## Author Summary

Ninety percent of schistosomiasis occurs in sub-Saharan Africa, where 91% of HIV-infected children reside. Both schistosomiasis and HIV affect the kidney, but their respective effects on kidney disease in children are not well described. Our prior work in HIV-infected adults demonstrated a high prevalence of kidney disease, possibly worsened by schistosomiasis, but adults are less commonly and less heavily infected with schistosomiasis than children. Therefore, we sought to describe the prevalence and factors associated with renal dysfunction (defined operationally as eGFR <60mL/min/1.73m^2^ and/or albuminuria >20mg/L in a single urine test) among HIV-infected children and their uninfected siblings living in a schistosomiasis endemic area. We found that half of HIV-infected children and more than one third of HIV-uninfected children had renal dysfunction. Schistosomiasis was the only factor significantly associated with renal dysfunction, increasing odds of renal dysfunction by 2.5-fold. Nearly 50% of the renal dysfunction we observed in both groups could be explained by schistosomiasis. The strong association between schistosomiasis and renal dysfunction among both HIV-infected and uninfected children should remind clinicians to screen for schistosomiasis. It also ought to spur further prospective research to assess for causality and outcomes in the relationship between *S. mansoni* and kidney disease in children.

## Introduction

HIV remains common in sub-Saharan Africa (SSA) where 91% of HIV-infected children reside and 1 in every 20 adults is infected [1, 2]. Kidney disease is an important complication in HIV-infected individuals and is associated with an increased risk of morbidity and mortality [3, 4]. The prevalence of kidney disease among HIV-infected adults in high-income countries ranges from 5%–50%, and is most common in patients of African descent [4]. Among more than 300 HIV-infected adults starting ART at our own hospital in Tanzania, 70% had evidence of kidney disease [5, 6].

Kidney disease among children with HIV is less well described [7]. In studies from SSA, the prevalence of markers of kidney disease among children with HIV varied greatly, ranging from 0–31.6%, depending on the methods used to evaluate the kidney [8–18]. In the few studies that included HIV-uninfected control subjects for comparison, the controls had less markers of kidney disease (0–6% versus 0–20.5%) [8, 9, 13–15]. None of these studies determined both estimated glomerular filtration rate (eGFR) and albuminuria. For this reason, the total prevalence of kidney disease among HIV-infected children in SSA remains unknown, and it is difficult to know if the kidney disease observed among children in these studies was due to HIV infection itself, medication use, or other factors that might be common among African children.

Therefore we conducted a cross-sectional study among HIV-infected children and their HIV-uninfected siblings in an area where kidney disease is common among HIV-infected adults. The objectives of our study were to determine the prevalence and correlates of renal dysfunction (defined operationally as eGFR <60mL/min/1.73m^2^ and/or albuminuria >20mg/L in a single urine test) and to compare the prevalence of renal dysfunction among HIV-infected and HIV-uninfected children. Our hypothesis was that the prevalence of undiagnosed renal dysfunction would be 30% and 5% among HIV-infected and uninfected children, respectively, and that active schistosome infection would be associated with renal dysfunction.

## Methods

### Trial design and study participants

This cross-sectional study was completed between August—December 2013 in the outpatient HIV clinic at Sekou Toure Regional Hospital. Sekou Toure Hospital is located in the city of Mwanza, along the shore of Lake Victoria in northwestern Tanzania, and it serves a population of approximately 2.7 million people. The outpatient HIV clinic of Sekou Toure follows approximately 475 children who are referred to the clinic from the surrounding community-based voluntary counseling and testing centers in Mwanza. In our study, we enrolled HIV-infected children ages 2–12 years old who were attending the Sekou Toure HIV clinic. All of the mothers of these HIV-infected children were also HIV-infected, so we assume that these children were perinatally infected. The caretakers of all enrolled HIV-infected children were invited to bring uninfected siblings between the ages of 2–12 years for enrollment as controls. We tested siblings for HIV using the Determine HIV-1/2 rapid antibody test (Alere Medical Co., Ltd, Chiba, Japan) as recommended by the Tanzanian National HIV Guidelines [19]. We excluded children with fever and those for whom urine samples could not be obtained.

### Data and sample collection

At least one parent or guardian for each child was interviewed. Both HIV-infected and uninfected children were examined. A structured questionnaire was used to collect demographic information, past medical history, and clinical symptoms. Additional information collected from HIV-infected children included a history of opportunistic infections, antiretroviral therapy (ART) status, and WHO clinical stage. In both groups (HIV-infected and uninfected siblings), 4 milliliters of blood were drawn at the time of enrollment to measure the random blood glucose and serum creatinine as well as CD4^+^ T-cell counts for HIV-infected children. Clean, midstream urine samples were collected for most children, except for a small group of children ≤ 4 years old in whom urine bags were used for urine collection. Urine samples were collected between 8–10AM, after ≥2 hours of fasting. Children ≤4 years old who did not urinate within 1 hour were excluded in order to minimize the inconvenience to their mothers. In order to determine the species of *Schistosoma* and intensity of infection in this population, we additionally obtained 10mL of urine and fresh fecal samples on 40 consecutive children who were CCA positive.

### Laboratory analyses

Random blood glucose was measured using a OneTouch® glucometer (LifeScan, Inc., Milpitas, California, USA). Fresh urine samples were tested immediately for albumin using a dipstick (Micral B, Roche, Mannheim, Germany). Patients were considered to have albuminuria if the urine albumin concentration was above 20 mg/L, as per instructions provided by the manufacturer and according to our prior research [5, 6, 20], and since a concentration of >20 mg/L has been demonstrated to correlate well with elevated albumin excretion rates by standard laboratory methods [21]. A urine dipstick (Multistix 10SG, Siemens, USA) was used to test for leukocyte esterase, hematuria, nitrates, glucose and ketones. The fresh urine sample was also tested using a circulating cathodic antigen (CCA) cassette test (Rapid Medical Diagnostics, Pretoria, South Africa) to detect active *Schistosoma* infection. The CCA test indicates active schistosome infection and can be positive in the urine during infection with either species of schistosomes that are endemic in Tanzania (*S. mansoni and S. haematobium*), though its sensitivity is lower in *S. haematobium* [22–24]. CCA point­of­care testing is used widely and has been found to be more sensitive than the gold standard Kato­Katz stool diagnosis of *S. mansoni*, particularly for lighter infections [25]. Following the manufacturer’s instructions, any visible line in the “test” area was considered positive. Line intensities were graded as “1” (test line very faintly visible), “2” (test line visible but lighter than control line), “3” (test line equal to control line), and “4” (test line darker than control line). For fecal samples, five slides were prepared using the Kato-Katz technique, as this has been shown to have a sensitivity comparable to examining stool specimens collected on different days [26]. Ten milliliters of fresh urine was filter concentrated and examined immediately by microscopy for both contamination and for *S. haematobium*. Intensity of infection was quantified as *S. mansoni* eggs per gram of stool and *S. haematobium* eggs per 10mL of urine. The laboratory personnel who performed these analyses were blinded to the HIV-status of the study subjects.

Serum creatinine was measured using a COBAS Integra 400 Plus clinical chemistry machine (Roche, Germany), calibrated by the Creatinine Jaffe 2 Method. An estimated glomerular filtration rate (eGFR) was calculated using the modified Schwartz equation as recommended by the Kidney Disease Improving Global Outcomes (KDIGO) guidelines [27].

### Definition

Renal dysfunction was defined operationally as an eGFR ≤60 ml/min/1.73 m^2^ and/or albuminuria >20 mg/L on a single urine dipstick. The severity of renal dysfunction was classified as Stage 1 (albuminuria + eGFR≥90), Stage 2 (albuminuria + eGFR 60–89), Stage 3 (eGFR 30–59), Stage 4 (eGFR 15–29) or Stage 5 (eGFR<15). Given the age of our study population (2 to 12 years), we defined malnutrition according to the BMI-for-age child growth standards from the WHO, defining severe malnutrition as a z-score of ≤-3 [28]. We also reported weight-for-age and height-for-age for those children <5 years old.

### Data analysis

The primary outcome was renal dysfunction as defined above. Based on the two-sample proportions Fisher’s exact test, we calculated that 122 children would be needed in each group to provide >95% power (at p = 0.05) to detect the difference in prevalence of renal dysfunction between the two groups that we hypothesized (30% and 5% among HIV-infected and uninfected children respectively) while also providing >80% power to show an association between schistosomiasis and renal dysfunction if the prevalence of schistosomiasis was 50% in the children with renal dysfunction and 25% in the children without renal dysfunction.

Data was entered into Microsoft Excel 2010 and analyzed using STATA version 12 (STATA Corporation, San Antonio, Texas). Categorical variables were described as proportions (%), and continuous variables were described as medians [interquartile range]. Univariable logistic regression analysis was performed to determine which baseline characteristics were associated with renal dysfunction. All variables significantly associated with renal dysfunction by univariable analysis were subjected to a predetermined multivariable logistic regression model, which also automatically included the variables for age, gender, and HIV status. The validity of the multivariable logistic regression model was assessed using the likelihood ratio test and by assessing for interactions. In addition, the linearity assumption was checked for continuous variables by comparing models with these variables represented continuously versus categorically. After schistosomal infection was found to be associated with renal dysfunction, we decided that we should perform an additional univariable logistic regression analysis to determine which specific markers of renal dysfunction were associated with schistosomiasis. P values of less than 0.05 were considered statistically significant.

### Ethical issues

Ethical approval for the study was obtained from the Research and Publications Committee of Bugando Medical Centre (under whose jurisdiction Sekou Toure Regional Hospital falls), as well as the Institutional Review Board of Weill Cornell Medical College. Informed written consent was obtained from the parents and assent was obtained from children ≥8 years. The results of all tests were reported immediately to the clinician caring for the child for the sake of further management. All children with schistosomiasis were treated according to the Tanzanian National Guidelines with 40 mg/kg of praziquantel.

## Results

### Study enrollment

During the study period, 139 HIV-infected children were seen at the Sekou Toure HIV clinic, and 17 were excluded for the following reasons: 8 were not able to provide urine samples, 5 had an acute, febrile illness, and 4 parents did not consent. In the end, 122 HIV-infected children were enrolled. For all enrolled HIV-infected children, parents were invited to bring HIV-uninfected siblings between the ages of 2–12 years for enrollment as controls. A total of 132 siblings were screened, and 10 were excluded for the following reasons: 6 were not able to provide urine samples, and 4 had an acute, febrile illness. A total of 122 siblings were enrolled. The characteristics of excluded children did not differ between the 2 groups.

### Baseline characteristics

Table 1 describes the baseline characteristics of the 2 groups. Among the 122 HIV-infected children, the median age was 8 years [4–11] and 63/122 (51.6%) were female. Among the 122 HIV-uninfected children, the median age was 8 years [5–10], and 55/122 (45.1%) were female.

**Table 1:** Baseline characteristics of HIV-infected children and HIV-uninfected siblings.

	**HIV-infected cases**	**HIV-uninfected siblings**	**p-value**
	**(n = 122)**	**(n = 122)**	
	**n (%) or median [IQR]**	**n (%) or median [IQR]**	
**Age (years)**	8 [4–11]	8 [5–10]	0.80
**Sex**			
Male	59 (48.4)	67 (54.9)	0.31
Female	63 (51.6)	55 (45.1)	
**Symptoms**			
Rash	12 (9.8)	6 (4.9)	0.14
Oral Sore	2 (1.6)	1 (0.8)	0.56
Diarrhea	1 (0.8)	1 (0.8)	1.0
Cough	26 (21.3)	12 (9.8)	**0.01**
Edema	0 (-)	0 (-)	-
**Past Medical History**			
Tuberculosis	10 (8.2)	1 (0.8)	**0.005**
Herpes Zoster	3 (2.5)	0 (-)	0.08
Recurrent Pneumonia	11 (9.0)	3 (2.5)	**0.03**
Oral Thrush	9 (7.4)	4 (3.3)	15
Papular Pruritic Eruptions	12 (9.8)	2 (1.6)	**0.01**
Known Kidney Disease	0 (-)	0 (-)	-
**Co-trimoxazole Use**	58 (52.3)	NA	-
**Physical Examination**			
Pallor	10 (8.2)	2 (1.6)	**0.02**
Thrush	4 (3.3)	0 (-)	**0.04**
Lymphadenopathy	23 (18.9)	3 (2.5)	**<0.001**
**CD4 Count (cells/uL)**	521.5 [302–828]	NA	-
**Malnutrition**			
BMI-for-age Z-scores	-0.17 [-1.01–1.26]	0.17 [-0.67–0.99]	0.67
Severe malnutrition*	1 (0.8)	2 (1.6)	0.57
**WHO Clinical Staging**			
1	3 (2.5)	NA	-
2	39 (32.2)	NA	-
3	67 (55.4)	NA	-
4	12 (9.9)	NA	-
Random Blood Glucose (mmol/L)	5.8 [4.9–6.7]	6 [5.2–6.8]	0.61

*Severe malnutrition has been defined as a BMI-for-age Z score ≤-3.

Historical factors that were significantly different between the 2 groups included cough (26/122 [21.3%] vs. 12/122 [9.8%], p = 0.01), history of tuberculosis (10/122 [8.2%] vs. 1/122 [0.8%], p = 0.005), recurrent pneumonia (11/122 [9.0%] vs. 3/122 [2.5%], p = 0.03), and papular pruritic eruptions (12/122 [9.8%] vs. 2/122 [1.6%], p = 0.01). Physical exam factors that were significantly different between the groups were pallor (10/122 [8.2%] vs. 2/122 [1.6%], p = 0.02), thrush (4/122 [3.3%] vs. 0/122 [0%], p = 0.04) and lymphadenopathy (23/122 [18.9%] vs. 3/122 [2.5%] p=<0.0001). No children reported history of neurologic disease and none had signs of neurologic disease on physical examination. The BMI-for-age z-scores were similar in the 2 groups. For the children <5 years old, the weight-for-age and height-for-age z-scores were also similar in the 2 groups (-0.76 [-1.57–0.05] vs. -0.48 [-1.12–0.21], p = 0.47 and -1.58 [-2.16–0.13] vs. -1.33 [-2.54–0.49], p = 0.47 respectively).

### Renal dysfunction outcomes

Table 2 describes the renal dysfunction outcomes (defined operationally as eGFR <60mL/min/1.73m^2^ and/or albuminuria >20mg/L in a single urine test). Among 122 HIV-infected children, 61/122 (50.0%) met our criteria for renal dysfunction: 54 (44.3%) had albuminuria, and 9 (7.4%) had an eGFR <60. Among 122 HIV-uninfected children, 51 (41.8%) met our criteria for renal dysfunction: 48 (39.3%) had albuminuria and 6 (4.9%) had an eGFR < 60.

**Table 2:** Markers of renal dysfunction in HIV-infected children and HIV-uninfected siblings.

	**HIV-infected cases**	**HIV-uninfected siblings**	**p-value**
	**(n = 122)**	**(n = 122)**	
	**n (%) or median [IQR]**	**n (%) or median [IQR]**	
Renal dysfunction*	61 (50.0)	51 (41.8)	0.20
**eGFR^†^ (ml/min/1.73 m^2^)**	112.9 [84.9–143–6]	113.3 [91.4–134.2]	1.0
**eGFR Category**			
Stage 1 (≥ 90)	88 (72.1)	95 (77.9)	0.43
Stage 2 (60–89)	25 (20.5)	21 (17.2)	
Stage 3 (30–59)	9 (7.4)	5 (4.1)	
Stage 4 (15–29)	0 (-)	0 (-)	
Stage 5 (< 15)	0 (-)	1 (0.8)	
**Albuminuria**			
Negative	68 (55.7)	74 (60.7)	0.88
>20 mg/L	38 (31.2)	35 (28.7)	
*>*50 mg/L	11 (9.0)	9 (7.4)	
*>*100 mg/L	5 (4.1)	4 (3.3)	
**Hematuria**	12 (9.8)	13 (10.6)	0.83
**Pyuria**	8 (6.6)	6 (4.9)	0.58

*the primary study outcome (defined operationally as eGFR <60mL/min/1.73m^2^ and/or albuminuria >20mg/L in a single urine test)

^†^eGFR = estimated glomerular filtration rate by modified Schwartz equation

### Factors associated with renal dysfunction

Table 3 shows the results of the univariable analysis for factors associated with renal dysfunction. In the univariable analysis only the presence of schistosomiasis was significantly associated with renal dysfunction (OR = 2.51, 95%CI 1.46–4.31, p = 0.001). Higher intensity of schistosomiasis was associated with higher prevalence of renal dysfunction (OR = 1.3 (1+) vs. OR≈4 (2+/3+), p for trend = 0.001). By multivariable logistic regression analysis including schistosomiasis, age, sex and HIV status, schistosomiasis remained the only factor associated with renal dysfunction (OR = 2.40, 95%CI 1.37–4.17, p = 0.002). HIV infection was not significantly associated with renal dysfunction by either univariable or multivariable analysis. There was more renal dysfunction among children with higher WHO clinical stage, but this relationship was not statistically significant (p = 0.23). Of note, though, the association between schistosomiasis and renal dysfunction was somewhat stronger among HIV-infected children than their HIV-uninfected siblings (OR = 3.04, 95%CI 1.39–6.68, p = 0.005 vs. OR = 2.08, 95%CI 0.97–4.46, p = 0.06).

**Table 3:** Factors associated with renal dysfunction among HIV-infected children and HIV-uninfected siblings by univariable logistic regression.

	**With Renal Dysfunction**	**Without Renal Dysfunction**	**Odds Ratio (95% CI)**	**p-value**
	**(n = 112)**	**(n = 132)**		
	**n (%) or median [IQR]**	**n (%) or median [IQR]**		
**Age** (years)	9 [5–11]	8 [4.5–10]	1.50	0.22
**Sex**				
Male	57 (50.89)	61 (46.21)	0.83 (0.51–1.37	0.47
Female	55 (49.11)	71 (53.79)		
**Symptoms**				
Rash	5 (4.46)	13 (9.85)	0.43 (0.15–1.24)	0.12
Oral Sore	1 (0.89)	2 (1.52)	0.59 (0.05–6.54)	0.66
Diarrhea	1 (0.89)	1 (0.76)	1.18 (0.07–19.09)	0.91
Cough	17 (15.18)	21 (15.91)	0.95 (0.47–1.90)	0.88
Edema	0 (-)	0 (-)	-	-
**Past Medical History**				
Fever	5 (4.46)	9 (6.82)	0.64 (0.21–1.96)	0.43
TB	8 (7.14)	3 (2.27)	3.30 (0.86–12.78)	0.08
Herpes Zoster	1 (0.89)	3 (2.27)	1.18 (0.07–19.09)	0.91
Recurrent Pneumonia	4 (3.57)	2 (1.52)	2.41 (0.43–13.40)	0.32
Oral Thrush	0 (-)	3 (2.27)	-	-
**Co-trimoxazole use**	33 (29.46)	33 (25.00)	1.25 (0.71–2.21)	0.43
**Physical Examination**				
Pallor	5 (4.5)	7 (5.3)	0.83 (0.26–2.71)	0.76
Thrush	1 (0.9)	3 (2.3)	0.39 (0.04–3.78)	0.41
Lymphadenopathy	16 (14.3)	10 (7.6)	2.03 (0.88–4.68)	0.10
Severe Malnutrition	1 (0.9)	2 (1.5)	0.59 (0.05–6.54)	0.66
CD4 count (cells/uL)*				
Mean	535.8 (399.3)	644.7 (363.4)	1.58	0.11
Median	481 [204–747]	612 [341–895)	0.82	0.37
WHO Clinical Stage*				
1	0 (-)	3 (4.9)	-	
2	16 (26.7)	23 (37.7)	0.35 (0.09–1.35)	
3	36 (60.0)	31 (50.8)	0.58 (0.16–2.12)	0.23
4	8 (13.3)	4 (6.6)	1	
**Random Blood Glucose**	6.2 [5.2–6.8]	5.8 [5–6.7]	2.25	0.13
***Schistosoma* positive^†^**	51 (45.5)	33 (25.00)	2.51 (1.46–4.31)	**0.001**
***Schistosoma* intensity^†^**				
Negative	61 (54.5)	99 (75.0)	1	
1+	12 (10.7)	15 (11.4)	1.30 (0.57–2.96)	
2+	13 (11.6)	5 (3.8)	4.22 (1.43–12.4)	**0.001**
3+	26 (23.2)	13 (9.9)	3.25 (1.55–6.79)	
**HIV-infected**	61 (54.5)	61 (46.2)	1.39 (0.84–2.31)	0.19

*For the 122 HIV-infected cases only

^†^ *Schistosoma* infection and intensity were both determined with the urine CAA assay.

### Renal dysfunction and *Schistosoma* infection

Table 4 compares the prevalence of renal dysfunction among *Schistosoma* infected children and *Schistosoma* uninfected children regardless of HIV status. An eGFR less than 60 ml/min/1.73m^2^ was more common in the *Schistosoma* infected children (7.2% versus 5.6%, OR 2.61, 95% CI 1.35–5.04, p = 0.01). There was also a higher prevalence of albuminuria among *Schistosoma* infected children (55.9% versus 34.4%, OR 2.92, 95% CI 1.61–5.27, p = 0.001). The overall prevalence of renal dysfunction was 60.7% among *Schistosoma* infected children and 38.1% among uninfected children (OR 2.51, 95% CI 1.46–4.31, p = 0.001).

**Table 4:** Markers of renal dysfunction associated with *Schistosoma* infection by univariable logistic regression.

	***Schistosoma* +ve**	***Schistosoma—*ve**	**Odds ratio (95%CI)**	**p-value**
	**(n = 84)**	**(n = 160)**		
	**Number (%) or Median [IQR]**	**Number (%) or Median [IQR]**		
Renal dysfunction*	51 (60.7)	61 (38.1)	2.51 (1.46–4.31)	**0.001**
**eGFR^†^ (ml/min/1.73m^2^)**	102.7 [77.5–131.7]	115.4 [92.9–141.8]	3.6	0.059
**eGFR <60ml/min/1.73m^2^**	6 (7.2)	9 (5.6)	2.61 (1.35–5.04)	**0.014**
**Albuminuria**	47 (55.9)	55 (34.4)	2.92 (1.61–5.27)	**0.001**
**Hematuria**	14 (16.7)	11 (6.9)	2.71 (1.17–6.27)	**0.02**
**Pyuria**	8 (4.9)	6 (7.1)	2.70 (0.91–8.06)	0.07

*the primary study outcome (defined operationally as eGFR <60mL/min/1.73m^2^ and/or albuminuria >20mg/L in a single urine test)

^†^eGFR = estimated glomerular filtration rate as calculated by modified Schwartz equation

### Schistosome species

None of the 40 subjects who provided urine for microscopy had eggs of *S. haematobium* detectable by microscopy. Of the 7 subjects from whom stool samples were available, 6/7 (85.7%) had *S. mansoni* eggs detected, with concentrations ranging from 17 to 75 eggs per gram.

## Discussion

In our study, half of the HIV-infected children attending an HIV clinic in the Lake Zone of northwestern Tanzania had evidence of renal dysfunction (defined operationally as eGFR <60mL/min/1.73m^2^ and/or albuminuria >20mg/L in a single urine test): 44.3% had albuminuria >20mg/L and 7.4% had an eGFR <60 ml/min/1.73 m^2^. These rates are higher than those found in other studies from SSA. Four other countries in SSA (Burkina Faso, Democratic Republic of Congo, Nigeria, and Zimbabwe) have reported the prevalence of markers of kidney disease among HIV-infected children as ranging from 0–31.6% using methodologies similar to ours [8–18]. The reasons for the higher prevalence of renal dysfunction among HIV-infected children in the Lake Zone compared to prior studies in SSA is not known, but our findings are consistent with the findings among HIV-infected adults in our region [5, 6].

Surprisingly, the prevalence of renal dysfunction was equally high among HIV-uninfected siblings. More than one-third of HIV-uninfected siblings had evidence of renal dysfunction: 39.3% had albuminuria >20 mg/L and 4.9% had eGFR<60 ml/min/1.73 m^2^. In other studies among children in SSA that have included HIV-uninfected controls, the prevalence of markers of kidney disease in the control group has been low (0–6%) [8, 9, 13–15]. The higher prevalence of renal dysfunction among HIV-uninfected children in our study is likely related to the unique nature of our control group. In order to minimize differences in socioeconomic factors, household exposures and genetics, we chose HIV-uninfected siblings as our control group, whereas prior studies have all used children from the pediatric outpatient clinics of the hospital in which the study was conducted as their controls [8, 9, 13–15]. The high prevalence of renal dysfunction that we observed among HIV-uninfected controls could therefore be related to either in utero HIV exposure or a high population prevalence of renal dysfunction. Because the dates of maternal HIV infection and viral suppression were not known, we could not confirm the HIV-exposure status among HIV-uninfected control siblings, but we suspect that most if not all were exposed since they were born within ~5 years of their HIV-infected sibling. HIV exposure, even in the absence of infection, has been associated with multiple abnormalities that may affect the development and function of the kidney in childhood [29–31]. On the other hand, the high prevalence of renal dysfunction among controls could also reflect a high community prevalence of renal dysfunction among children, possibly related to the known high prevalence of schistosomiasis. In order to investigate this possibility, we are currently planning a longitudinal study to examine a cohort of HIV-uninfected, unexposed school children in the Lake Zone.

Schistosomiasis was strongly associated with renal dysfunction among HIV-infected and uninfected children (OR = 2.51, 95% CI 1.46–4.31), and a higher intensity schistosome infection was associated with even more renal dysfunction (p = 0.001 for trend). Approximately 46% of children with renal dysfunction had schistosomiasis by CCA testing compared to 25% of children without renal dysfunction. Multiple studies and epidemiological evidence have shown that *S. haematobium* infection may cause kidney disease (particularly albuminuria) in both adults and children [32–34], but all of the 40 consecutive urine samples we tested were negative for *S. haematobium*, and <20% of CCA-positive subjects had the hematuria that is typical for this infection. The dominant species of schistosomiasis in our region, *S. mansoni* [35, 36], is also known to cause glomerulonephritis and kidney disease [37]. The prevalence of proteinuria among adult subjects infected with *S. mansoni* has been reported to be as high as 20% in Egypt [38, 39] and 15% in Brazil [40, 41], and the severity and irreversibility of the disease has been demonstrated in several clinicopathologic and experimental studies [42–44]. The association between markers of kidney disease and *S. mansoni* in children, by contrast, has only been investigated in two small studies in SSA [45, 46] which did not find an association between *S. mansoni* and overt proteinuria, but neither one of them investigated both eGFR and albuminuria as we have done. If further studies confirm the relationship between *S. mansoni* infection and kidney disease among children, schistosomiasis may become an important target for prevention of kidney disease in our population.

Although schistosomiasis could explain a large proportion of the renal dysfunction in our study, 55% of children with renal dysfunction did not have evidence of current schistosomiasis. In these subjects multiple other factors may be contributing to their renal dysfunction such as acute glomerulonephritis, infections (malaria, recurrent diarrhea), or genetic factors. The APOL1 gene mutation, for example, is known to be associated with renal disease in African populations [47], younger age of onset of kidney disease [48] and faster decline in kidney function [49]. Further studies are needed to determine the factors other than schistosomiasis that might be contributing to the high prevalence of renal dysfunction that we observed. Kidney disease occurring at a young age may lead to end stage renal disease or other complications (e.g. hypertension) in adulthood; therefore targeted screening for early detection of kidney disease and treatment of reversible factors are high priorities. Many simple diagnostic tools such as light microscopy of the urine sediment are currently underutilized in screening efforts. In addition, the implementation of proven strategies for prevention and treatment, such as early antimicrobial therapy for severe infections and rapid correction of hypovolemic shock, must be accelerated.

Our study has several limitations. First and foremost, this was a cross-sectional study and, therefore, neither confirmation of chronic kidney disease nor causality in the relationship between schistosomiasis and kidney disease could be examined. In addition, several gold standard investigations for HIV and kidney disease, such as quantitative HIV viral load testing, urine albumin-to-creatinine ratio, and kidney biopsies were not performed since they were not available in our region at the time of the study. Our operational definition of renal dysfunction may have resulted in information bias, with over diagnosis of kidney disease, and we are currently planning to assess our findings with a study using the standard KDIGO definition of chronic kidney disease. Also, the exclusion of children who could not produce urine samples, as well as the possibility that HIV-infected patients may have received previous praziquantel therapy could have caused some selection bias and underestimation of the population prevalences of renal dysfunction and schistosomiasis, respectively.

In conclusion, our study identified a high prevalence of renal dysfunction (defined operationally as eGFR <60mL/min/1.73m^2^ and/or albuminuria >20mg/L in a single urine test) among HIV-infected Tanzanian children attending our pediatric HIV clinics. Almost 50% of both HIV-infected children and their siblings had renal dysfunction, and 6% had an eGFR <60 mL/min/1.73 m^2^. Surprisingly, the prevalence of renal dysfunction among HIV-uninfected siblings was similar to the HIV-infected children. This may be related to either in utero HIV exposure or a high community-wide prevalence of renal dysfunction in children, and further studies are urgently needed to distinguish these possibilities. Schistosomiasis was strongly associated renal dysfunction in this population, and the predominant species of schistosomes in our region is *S. mansoni* (not *S. haematobium)*. Schistosomiasis may be an important target for prevention of kidney disease in children in sub-Saharan Africa.

## Supporting Information

S1 Checklist Completed STROBE checklist for cross-sectional studies. (DOCX)
