# Supplementary material for: The Influence of HIV and Schistosomiasis on Renal Function: A Cross-sectional Study among Children at a Hospital in Tanzania
Source: PLoS Negl Trop Dis. 2015 Jan 22;9(1):e0003472. doi: 10.1371/journal.pntd.0003472 (PMC4303314; doi:10.1371/journal.pntd.0003472)
Supplement: S1 Checklist — (DOCX) [file pntd.0003472.s001.docx]

STROBE Statement—checklist of items that should be included in reports of observational studies

|  | Item No. | Recommendation | Page  No. | Relevant text from manuscript |
| --- | --- | --- | --- | --- |
| **Title and abstract** | 1 | (*a*) Indicate the study’s design with a commonly used term in the title or the abstract | 1 | Title: HIV, schistosomiasis and renal dysfunction in children: a cross-sectional study at a Tanzanian HIV clinic |
|  |  | (*b*) Provide in the abstract an informative and balanced summary of what was done and what was found | 2 | see abstract |
| Introduction | | | |  |
| Background/rationale | 2 | Explain the scientific background and rationale for the investigation being reported | 4 |  |
| Objectives | 3 | State specific objectives, including any prespecified hypotheses | 4 | The objectives of our study were to determine the prevalence and correlates of renal dysfunction and to compare the prevalence of renal dysfunction among HIV-infected and HIV-uninfected children. Our hypothesis was that the prevalence of undiagnosed renal dysfunction would be 30% and 5% among HIV-infected and uninfected children, respectively, and that active schistosome infection would be associated with renal dysfunction. |
| Methods | | | |  |
| Study design | 4 | Present key elements of study design early in the paper | 5 |  |
| Setting | 5 | Describe the setting, locations, and relevant dates, including periods of recruitment, exposure, follow-up, and data collection | 5-6 |  |
| Participants | 6 | (*a*) *Cohort study*—Give the eligibility criteria, and the sources and methods of selection of participants. Describe methods of follow-up  *Case-control study*—Give the eligibility criteria, and the sources and methods of case ascertainment and control selection. Give the rationale for the choice of cases and controls  ***Cross-sectional study*—Give the eligibility criteria, and the sources and methods of selection of participants** | 5 | We enrolled HIV-infected children ages 2 - 12 years old who were attending the Sekou Toure HIV clinic.  The caretakers of all enrolled HIV-infected children were invited to bring uninfected siblings between the ages of 2 - 12 years for enrollment as controls. We tested siblings for HIV using the Determine HIV-1/2 rapid antibody test (Alere Medical Co., Ltd, Chiba, Japan) as recommended by the Tanzanian National HIV Guidelines. We excluded children with fever and those for whom urine samples could not be obtained. |
|  |  | (*b*) *Cohort study*—For matched studies, give matching criteria and number of exposed and unexposed  *Case-control study*—For matched studies, give matching criteria and the number of controls per case | n/a |  |
| Variables | 7 | Clearly define all outcomes, exposures, predictors, potential confounders, and effect modifiers. Give diagnostic criteria, if applicable | 6-7 | See methodology subsections laboratory analyses and definitions. |
| Data sources/ measurement | 8* | For each variable of interest, give sources of data and details of methods of assessment (measurement). Describe comparability of assessment methods if there is more than one group | 6-7 | See methodology subsections laboratory analyses and definitions. |
| Bias | 9 | Describe any efforts to address potential sources of bias | 7 | blinding of laboratory personnel |
| Study size | 10 | Explain how the study size was arrived at | 7 | Based on the two-sample proportions Fisher's exact test, we calculated that 122 children would be needed in each group to provide >95% power (at p=0.05) to detect the difference in prevalence of renal dysfunction between the two groups that we hypothesized (30% and 5% among HIV-infected and uninfected children respectively) while also providing >80% power to show an association between schistosomiasis and renal dysfunction if the prevalence of schistosomiasis was 50% in the children with renal dysfunction and 25% in the children without renal dysfunction. |

Continued on next page

| Quantitative variables | 11 | Explain how quantitative variables were handled in the analyses. If applicable, describe which groupings were chosen and why | 8 | see 2^nd^ paragraph of data analysis section |
| --- | --- | --- | --- | --- |
| Statistical methods | 12 | (*a*) Describe all statistical methods, including those used to control for confounding | 7-8 | see data analysis |
|  |  | (*b*) Describe any methods used to examine subgroups and interactions |  |  |
|  |  | (*c*) Explain how missing data were addressed | n/a |  |
|  |  | (*d*) *Cohort study*—If applicable, explain how loss to follow-up was addressed  *Case-control study*—If applicable, explain how matching of cases and controls was addressed  *Cross-sectional study*—If applicable, describe analytical methods taking account of sampling strategy | n/a |  |
|  |  | (*e*) Describe any sensitivity analyses | n/a |  |
| Results | | | | |
| Participants | 13* | (a) Report numbers of individuals at each stage of study—eg numbers potentially eligible, examined for eligibility, confirmed eligible, included in the study, completing follow-up, and analysed | 9 | see “study enrolment” |
|  |  | (b) Give reasons for non-participation at each stage | 9 | see “study enrolment” |
|  |  | (c) Consider use of a flow diagram | n/a |  |
| Descriptive data | 14* | (a) Give characteristics of study participants (eg demographic, clinical, social) and information on exposures and potential confounders | 9 | see “baseline characteristics” and Table 1 |
|  |  | (b) Indicate number of participants with missing data for each variable of interest | n/a |  |
|  |  | (c) *Cohort study*—Summarise follow-up time (eg, average and total amount) |  |  |
| Outcome data | 15* | *Cohort study*—Report numbers of outcome events or summary measures over time |  |  |
|  |  | *Case-control study—*Report numbers in each exposure category, or summary measures of exposure |  |  |
|  |  | *Cross-sectional study—*Report numbers of outcome events or summary measures | 10 | see “renal dysfunction outcomes” and Table 2 |
| Main results | 16 | (*a*) Give unadjusted estimates and, if applicable, confounder-adjusted estimates and their precision (eg, 95% confidence interval). Make clear which confounders were adjusted for and why they were included | 9-10 | see results section |
|  |  | (*b*) Report category boundaries when continuous variables were categorized | 7 | see laboratory analyses and definition |
|  |  | (*c*) If relevant, consider translating estimates of relative risk into absolute risk for a meaningful time period | n/a |  |

Continued on next page

| Other analyses | 17 | Report other analyses done—eg analyses of subgroups and interactions, and sensitivity analyses |  |  |
| --- | --- | --- | --- | --- |
| Discussion | | | | |
| Key results | 18 | Summarise key results with reference to study objectives | 12-15 | see discussion |
| Limitations | 19 | Discuss limitations of the study, taking into account sources of potential bias or imprecision. Discuss both direction and magnitude of any potential bias | 14 | last paragraph |
| Interpretation | 20 | Give a cautious overall interpretation of results considering objectives, limitations, multiplicity of analyses, results from similar studies, and other relevant evidence | 12-15 | especially see conclusion |
| Generalisability | 21 | Discuss the generalisability (external validity) of the study results | 14 | last paragraph |
| Other information | |  | | |
| Funding | 22 | Give the source of funding and the role of the funders for the present study and, if applicable, for the original study on which the present article is based | included in the online submission system | This project was supported in part by grants from the United States National Institute of Health/National Institute of Allergy and Infectious Diseases (AI098627), the National Institutes of Health Fogarty International Center (R25TW009337) and a United States Agency for International Development (USAID) leadership training program. The funders had no role in study design, data collection and analysis, decision to publish, or preparation of the manuscript |

*Give information separately for cases and controls in case-control studies and, if applicable, for exposed and unexposed groups in cohort and cross-sectional studies.

**Note:** An Explanation and Elaboration article discusses each checklist item and gives methodological background and published examples of transparent reporting. The STROBE checklist is best used in conjunction with this article (freely available on the Web sites of PLoS Medicine at http://www.plosmedicine.org/, Annals of Internal Medicine at http://www.annals.org/, and Epidemiology at http://www.epidem.com/). Information on the STROBE Initiative is available at www.strobe-statement.org.
